# Biomarkers for Immunotherapy of Oral Squamous Cell Carcinoma: Current Status and Challenges

**DOI:** 10.3389/fonc.2021.616629

**Published:** 2021-03-08

**Authors:** Alhadi Almangush, Ilmo Leivo, Antti A. Mäkitie

**Affiliations:** ^1^ Department of Pathology, University of Helsinki, Helsinki, Finland; ^2^ Research Program in Systems Oncology, Faculty of Medicine, University of Helsinki, Helsinki, Finland; ^3^ Department of Oral and Maxillofacial Diseases, University of Helsinki, Helsinki, Finland; ^4^ Institute of Biomedicine, Pathology, University of Turku, Turku, Finland; ^5^ Faculty of Dentistry, University of Misurata, Misurata, Libya; ^6^ Department of Pathology, Turku University Central Hospital, Turku, Finland; ^7^ Department of Otorhinolaryngology—Head and Neck Surgery, University of Helsinki and Helsinki University Hospital, Helsinki, Finland; ^8^ Division of Ear, Nose and Throat Diseases, Department of Clinical Sciences, Intervention and Technology, Karolinska Institutet and Karolinska University Hospital, Stockholm, Sweden

**Keywords:** oral squamous cell carcinoma, immunotherapy, biomarkers, immune response, survival

## Abstract

Oral squamous cell carcinoma (OSCC) forms a major health problem in many countries. For several decades the management of OSCC consisted of surgery with or without radiotherapy or chemoradiotherapy. Aiming to increase survival rate, recent research has underlined the significance of harnessing the immune response in treatment of many cancers. The promising finding of checkpoint inhibitors as a weapon for targeting metastatic melanoma was a key event in the development of immunotherapy. Furthermore, clinical trials have recently proven inhibitor of PD-1 for treatment of recurrent/metastatic head and neck cancer. However, some challenges (including patient selection) are presented in the era of immunotherapy. In this mini-review we discuss the emergence of immunotherapy for OSCC and the recently introduced biomarkers of this therapeutic strategy. Immune biomarkers and their prognostic perspectives for selecting patients who may benefit from immunotherapy are addressed. In addition, possible use of such biomarkers to assess the response to this new treatment modality of OSCC will also be discussed.

## Introduction

Survival rate of oral squamous cell carcinoma (OSCC) is about 50% of affected cases. Advances in traditional treatments (surgery, radiotherapy, chemotherapy) of OSCC have failed to increase survival and, at the same time, they have been associated with significant side effects. Prediction of survival in oral cancer depends on classical parameters such as tumor grade and depth of invasion, although many biomarkers have been introduced as potential prognosticators of OSCC (1, 2).

Recent research has introduced immunotherapy as an effective treatment option for OSCC. The hypothesis of immunotherapy was based on a theory that was introduced for more than a century ago postulating an ability of the immune system to repress cancer cells and aid in patient recovery (3). The significance of cancer immunotherapy was recognized more universally when the Nobel Prize in Physiology or Medicine was awarded for the development of such therapies in 2018 (4). For OSCC, immunotherapy was firstly approved for recurrent/metastatic cases (similar to other cancers of head and neck region) (5). Of note, neoadjuvant immunotherapy administered preoperatively has been recently introduced for untreated OSCC (6).

With the success of immunotherapy in the treatment of OSCC, it has become important to find parameters to select patients who might benefit from this treatment strategy as well as to find a predictive marker/s for following treatment response. In this mini-review we will discuss different methods that have been introduced to assess the immune response and immune biomarkers in OSCC.

## Assessment of Immune Response as a Part of Grading Systems of OSCC

Immune cells are among the main cellular components of cancer stroma tissue (7). The interaction of immune cells with tumor cells has been widely studied as one of the factors that influence tumor progression (8, 9). It has been reported in many cancers that active antitumor immune response is a feature of good prognosis (9, 10). Many proposals have suggested to assess the immune response as a part of histopathologic grading of OSCC. For example, an early study by Anneroth et al. (11) suggested to incorporate the assessment of the inflammatory cell infiltrate as a part of their malignancy grading system (11). They scored lymphoplasmacytic infiltrates into four categories as marked, moderate, slight or none (11). That system was modified later by Bryne et al. (12) who assessed malignancy grade (including the lymphoplasmacytic infiltrate) at the invasive front of OSCC (12). Brandwein-Gensler et al. (13) assessed the immune response as a part of a histologic risk score including three parameters: worst pattern of invasion, perineural invasion and lymphocytic host response (13). Most recently (2020), Bjerkli et al. proposed a histo-score based on the assessment of the lymphocytic infiltrate and tumor differentiation, and showed that the score gave a good prediction of survival in oral tongue cancer (14). Our group (15) proposed stromal classification, based on the assessment of tumor-infiltrating lymphocytes and tumor-stroma ratio, with a promising prognostic value in early oral tongue cancer.

From the above historically accumulated evidence, it seems that incorporation of the immune response as a part of the grading system of OSCC is a useful and important step which has not yet been implemented in pathology practice. A clinically relevant grading system with a robust association with tumor behavior and outcome, which considers the immune response is expected to become very useful for future immunotherapy of OSCC.

## Histologic Semiquantitative Assessment of Tumor-Infiltrating Lymphocytes

Morphological evaluation of tumor-infiltrating lymphocytes (TILs), using routine hematoxylin eosin (HE)-stained tumor sections, has been reported in many cancers including OSCC (16). A standardized method for the assessment of TILs has been introduced by the International TILs Working Group (9). Accumulating evidence has shown the significance of this method in various cancers (17–19). In OSCC, our group (15) has recently reported that the assessment of stromal TILs [as proposed by TILs Working Group (9)] can be used as a significant prognostic tool for the prediction of overall survival, disease-specific survival and disease-free survival in a large multicenter cohort of early oral tongue cancer. This assessment method has also been used successfully for other subsites of head and neck cancers (16). After further validation in large cohorts, this simple method for the assessment of TILs can be used to monitor response to immunotherapy. In addition to validation, it is important to overcome some limitations such as lack of consensus on the morphologic evaluation of TILs in OSCC and difficulty in assessing TILs using the preoperative diagnostic biopsies (20).

## Progress of Research on Immune Biomarkers of OSCC

In order to predict cancer response to immunotherapy, recent research (21) has tried to identify the immune profile of tumors classified into cold tumor (also known as immune desert) or hot tumor (also known as inflamed tumor). Using immunohistochemistry, several researchers have studied immune checkpoint molecules and the expression of specific TILs to identify the immune profile of OSCC. The mechanisms of such immune molecules were described in other articles (22, 23). Because so many studies have been published on immune biomarkers, we will focus here on the accumulated evidence from systematic reviews and meta-analyses. For example, Sievilainen et al. (24) in their recent systematic review covering the period from 1985 to 2017, on the prognostic value of immune checkpoints of OSCC have noted that seven immune checkpoints (PD‐L1, FKBP51, B7‐H4, B7‐H6, ALHD1, IDO1, and B7‐H3) had been reported to have an association with worse survival. In a meta-analysis of the prognostic value of TILs in OSCC, Huang et al. (25) found that high infiltration of CD8+ TILs, CD45RO+ TILs and CD57+ TILs associated with good survival; while high infiltration of CD163+ and CD68+ macrophages had an association with poor prognosis. In another meta-analysis, Hadler-Olsen et al. (26) found that CD163+ M2 and CD57+ had a promising relationship with outcome in patients with OSCC. Findings from these systematic reviews and meta-analyses should be considered as a cornerstone for future research in identifying the clinically most relevant immune biomarkers. It is necessary to acknowledge that the above-mentioned findings were reported from studies including samples mainly from patients treated with surgery and other traditional strategies, such as radiotherapy and/or chemotherapy.

For head and neck cancer including OSCC, treatment with anti-programmed cell death-1 (anti-PD-1) and anti-programmed cell death ligand-1 (anti-PD-L1) antibodies are crucial in the currently approved immunotherapy (27, 28). To identify which cases are more likely to benefit from such treatment, many researchers have studied the two relevant biomarkers (i.e. PD-1 and PD-L1) using samples from patients treated with immunotherapy. As an example, expression of PD-L1 showed a significant association with response to durvalumab (an anti-PD-L1 antibody) in recent studies of head and neck cancer (5, 29).These studies found that a cutoff of 25% of cancer cells staining with PD-L1 is suitable to determine the patient´s response to durvalumab immunotherapy (5, 29). In another study on the anticancer activity of pembrolizumab-based immunotherapy, however, Chow et al. (30) suggested to consider scoring of PD-L1 in both cancer cells and immune cells with a cutoff point of 1%. Similarly, Emancipator et al. (31) reported that a “combined positive score”, which evaluates the ratio of the number of PD-L1-expressing cells (including cancer cells and immune cells) to the number of all viable cancer cells multiplied by 100, is a powerful tool in assessing the response to pembrolizumab.

In a phase 3 trial including 361 patients with recurrent HNSCC who received nivolumab, the patient survival was improved with this kind of immunotherapy (32). However, expression of PD-L1 was not that significant in the assessment of response to the treatment (32). This might highlight the difficulty in comparing the findings across the studies that have used PD-L1 as a predictive marker if the immunotherapeutic agents were different. In addition, it is important to take into consideration that the above-mentioned findings on PD-1 and/or PD-L1 were reported from studies that included different subsites of head and neck cancer with well-known variation in their clinical behavior. Therefore, further trials should consider specific studies on OSCC to confirm the usefulness of PD-1 and PD-L1 in predicting the response to immunotherapy. In addition, whether to evaluate the expression of PD-L1 in both cancer cells and immune cells or only in immune cells needs to be determined based on the future studies. Furthermore, methods other than immunohistochemistry to assess immune biomarkers, such as immune-related signature, should be tested in OSCC cases treated with immunotherapy as this method has showed a good predictive value to immunotherapy in other tumors (33, 34).

## Immunoscore for OSCC

Recent research efforts have introduced an immune-based assay known as immunoscore based on the assessment of a combination of immune biomarkers to identify the outcome of cancer (35). The most promising results with immunoscore have been reported in colorectal cancer where a scoring system for the quantification of CD3 and CD8 were standardized and showed a promising predictive power superior to TNM staging system (36) and showed successful results in phase 3 clinical trials (37). For oral carcinoma, identification of immune feature-based prognostic score has been recently introduced by Zhou et al. (38) who reported a promising prognostic value for an immunoscore based on the evaluation of CD3 in central areas and at invasive margins of OSCC; CD8, CD45RO, and FOXP3 in the central part of OSCC; FOXP3 and CD45RO at invasive margins of OSCC. However, the proposed immunoscore for OSCC will require further validation.

## Digital Pathology and Immune Biomarkers

Automated assessment of immune biomarkers has been widely studied in different cancers with successful performance (39–41). In OSCC, such assessment is still at an early stage as only few studies have reported on this concept. However, those few reports have shown promising findings. Shaban et al. (2019) reported a digital score for objective quantification of TILs that can successfully predict disease-free survival in OSCC and showed a better prognostic value than the manual assessment of TILs (42). Of note, this method of assessing TILs using whole-slide images of hematoxylin and eosin (HE)-stained sections was also successfully used in other cancers (43). In another recent study, Huang et al. (44) reported a promising value for digital image analysis of CD8 in a large cohort of tongue cancers. This approach of evaluating immune markers using digital analysis can be a simple tool to assess the immune response of OSCC and therefore validation studies are required.

## Other Factors to Assess Response to Immunotherapy

In addition to immune response and immune biomarkers, other existing factors including tumor mutational burden and mutational signatures might be associated with response to immunotherapy (45). Tumor mutational burden, referring to number of somatic mutations per coding area of a tumor genome, has shown a prognostic value in many cancers (46). Of note, recent research has showed that tumor mutational burden has a significant value in prediction of response to the immunotherapy (45). In a cohort including cases of head and neck cancer, Cristescu et al. (47) found that tumor mutational burden and T cell-inflamed gene expression profile can together predict the clinical responses to immunotherapy with pembrolizumab, and a longer survival was reported with higher levels of these two factors. Although pembrolizumab has been recommended for cases with high tumor mutational burden (≥ 10 mutations/megabase), some researchers have caveated against such universal threshold, and highlighted the fact that patients with cancer are often receiving cytotoxic chemotherapies that might cause higher level of tumor mutational burden (48). Thus, it is still necessary to determine the optimal cutoff point for tumor mutational burden in each tumor type to identify the group that might benefit from immunotherapy. In addition, it is necessary to take into consideration that the tumor immune microenvironment is characterized by a complexity that warrants assessment of the clinical response from different aspects, and the measurement of tumor mutational burden being one of them.

## Conclusions and Perspectives

In the rapidly evolving field of immunotherapy, identification of biomarkers to predict the immune response can make such a therapy one of the clinically effective treatments of OSCC. There are many parameters/biomarkers and methods that have been introduced during the last three decades for the assessment of immune response. Ongoing research efforts include use of immune response in grading of OSCC, and identification of an immunoscore for OSCC. A successful clinically relevant assessment of the immune response can be considered as a cornerstone in identifying patients who will benefit from immunotherapy and also for following up the treatment response. Evidence from recent collaborative studies and/or meta-analyses highlighting the importance of evaluation of TILs and other immune biomarkers as a robust tool reveal the status of the immune response and have a strong correlation with survival outcome. There is an urgent need for validation studies to confirm the findings on these biomarkers, thus, to aid in identification of an ideal biomarker/s to select OSCC cases that can benefit from immunotherapy and to assess the patient´s response. Digital assessment of immune biomarkers in OSCC are still at an early stage and require further research. Similarly, findings on the predictive value of tumor mutational burden and mutational signatures still require further research before they can be added in the personalized prediction of OSCC treatment response.

## Author Contributions

All authors listed have made a substantial, direct, and intellectual contribution to the work and approved it for publication.

## Conflict of Interest

The authors declare that the research was conducted in the absence of any commercial or financial relationships that could be construed as a potential conflict of interest.
